# Interstitial nephritis without glomerulonephritis in ANCA-associated vasculitis: a case series and literature review

**DOI:** 10.1007/s10067-022-06264-2

**Published:** 2022-06-27

**Authors:** Xuxia He, Yubing Wen, Rongrong Hu, Haiting Wu, Wei Ye, Cai Yue, Yan Qin, Peng Xia, Limeng Chen

**Affiliations:** 1 Department of Internal Medicine, Peking Union Medical College Hospital, Chinese Academy of Medical Sciences and Peking Union Medical College, Beijing, 100730 China; 2 Department of Nephrology, Peking Union Medical College Hospital, Chinese Academy of Medical Sciences and Peking Union Medical College, Beijing, 100730 China; 3 State Key Laboratory of Complex Severe and Rare Diseases, Peking Union Medical College Hospital, Chinese Academy of Medical Sciences and Peking Union Medical College, Beijing, 100730 China

**Keywords:** ANCA-associated vasculitis, Case reports, Interstitial nephritis, Literature review

## Abstract

The typical nephrological presentation of antineutrophil cytoplasmic antibody (ANCA)–associated vasculitis (AAV) is rapidly progressive glomerulonephritis. AAV-associated interstitial nephritis without apparent glomerular lesions was rare. We reported three local cases of AAV-associated interstitial nephritis without glomerulonephritis confirmed by renal biopsy. Then, a literature search was conducted in PubMed using free text words and MeSH terms related to “AAV and interstitial nephritis”. Fifteen cases were included, and their demographics, clinical manifestations, laboratory data, renal pathological features, and treatment response were summarized. AAV-associated interstitial nephritis usually affects elderly patients. The common symptoms include fever, arthralgias, and edema. These patients were mostly MPO-ANCA positive. Pathological lesions in the kidney showed diffuse infiltration of inflammatory cells, edema, tubulitis, and fibrosis in the interstitial area. Various immunosuppressive treatments, including glucocorticoids, immunosuppressants, and rituximab, were used, and most of the patients achieved clinical remission. AAV-associated interstitial nephritis is rare but shows a characteristic clinical phenotype, serological results, and pathogenic lesions. Immunosuppressive therapy showed good efficacy in these patients.

## Introduction 

Antineutrophil cytoplasmic antibody (ANCA)–associated vasculitis (AAV) refers to a category of systemic, necrotizing vasculitis. It predominantly affects the small blood vessels of the middle-aged and elderly population. AAV includes microscopic polyangiitis (MPA), granulomatosis with polyangiitis (GPA), eosinophilic granulomatosis with polyangiitis (EGPA), renal-limited AAV, and certain drug-induced vasculitis syndromes which may be ANCA specific for either myeloperoxidase (MPO-ANCA) or proteinase 3 (PR3-ANCA) [1]. Patients with AAV typically present with nonspecific symptoms such as fever, malaise, anorexia, weight loss, myalgias, and arthralgias [2, 3]. The main organs involved in AAV patients include the kidney, respiratory tract, and occasionally the heart and brain. Kidney involvement is common in AAV, especially in GPA and MPA [1]. A study concerning patients with GPA or MPA showed that MPO-ANCA was often associated with renal, skin, and lung manifestations [4]. The typical presentation of kidney involvement in AAV is rapidly progressive glomerulonephritis presenting as asymptomatic hematuria, a variable degree of proteinuria, and acute renal insufficiency. Various kidney biopsy findings include mild focal and segmental glomerulonephritis as well as diffuse necrotizing and crescentic glomerulonephritis [5–7]. Although most AAV patients have pauci-immune glomerulonephritis, some have atypical clinical manifestations, such as interstitial nephritis associated with vasculitis in the vasa recta without evidence of glomerulonephritis [8, 9]. Limited literature has reported cases of AAV-associated interstitial nephritis without any apparent glomerular lesions.

In this narrative review, we report a case series of three AAV patients with interstitial nephritis without glomerulonephritis and compare it with six local cases of AAV-associated glomerulonephritis. Furthermore, we also reviewed the literature to study the clinical manifestation, pathological features, and treatment response of this kind of atypical AAV patient.

## Materials and methods

### Participants

**Fig. 1:**
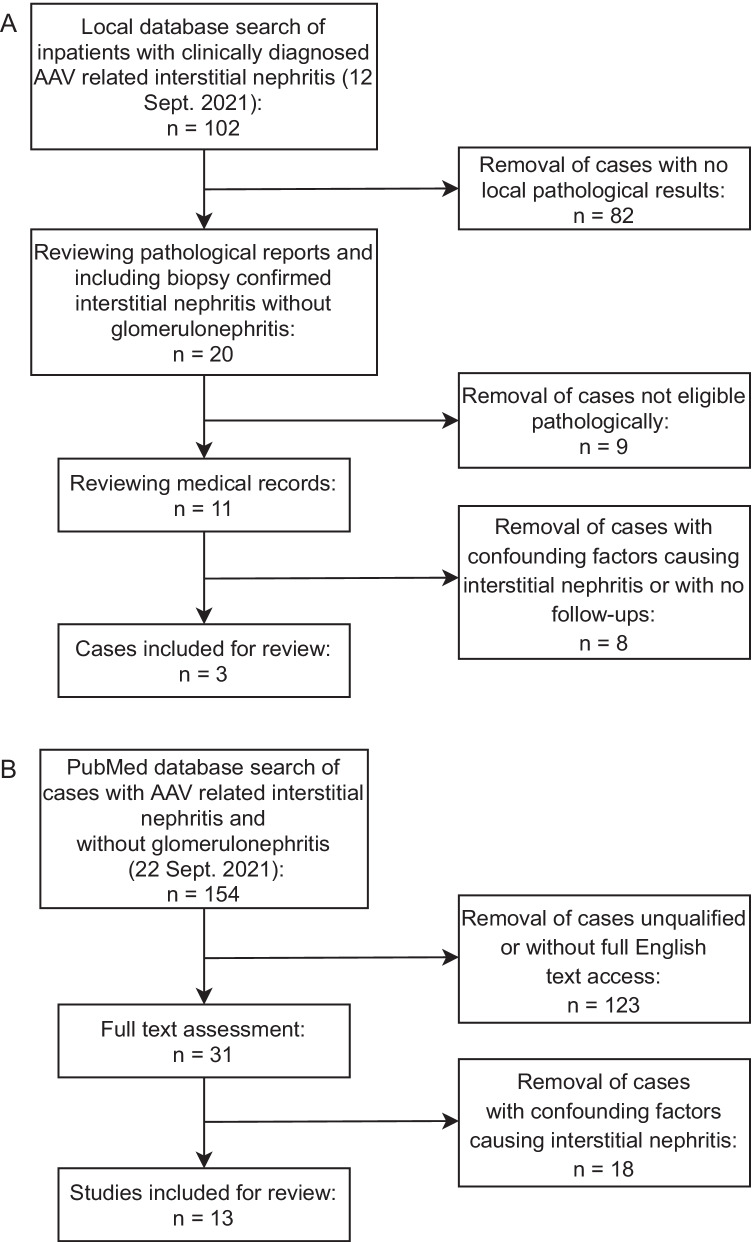
Flow chart of the research process incorporating three local cases (**A**) and thirteen cases at PubMed (**B**). AAV, antineutrophil cytoplasmic antibody–associated vasculitis

Patients diagnosed with AAV and interstitial nephritis without glomerulonephritis confirmed by renal biopsy admitted to Peking Union Medical College Hospital (PUMCH) from Jan. 2013 to Aug. 2021 were included in this study. Two nephrology pathologists reviewed their detailed renal pathology database. We recorded their demographics, clinical manifestations, laboratory data, renal pathological features, and treatment response. Patients with confounding factors potentially leading to interstitial nephritis, such as drug use, were excluded (Fig. 1A). A total of 3 cases were identified. No other significant organ involvement was noted in these cases. The data of the patients diagnosed with typical ANCA-associated glomerulonephritis were reviewed, and 6 patients were matched in terms of sex and age with the patients with AAV-associated interstitial nephritis. A *t* test was used to compare the two groups, and *P* < 0.05 was considered significant. This study was approved by the Ethical Committee of PUMCH (No. S-K 1877).

### Literature review

We searched the literature in PubMed with free text words and MeSH terms related to AAV and interstitial nephritis. The final search strategy was (interstitial nephritis [MeSH Terms] OR tubulointerstitial nephritis) AND ((anti-neutrophil cytoplasmic antibody-associated vasculitis [MeSH Terms]) OR (ANCA vasculitis OR microscopic polyangiitis OR granulomatosis OR eosinophilic granulomatosis OR Churg-Strauss)). All studies published before Sep. 22, 2021, were included for screening by title and abstract. Studies with overlapping data, not written in English, and without full-text access were excluded (Fig. 1B). The demographics, clinical manifestation, laboratory data, renal pathological features, and treatment response are summarized in Tables 1 and 2.

**Table 1:** Summary of 3 local cases and 15 reported cases with ANCA-associated vasculitis causing interstitial nephritis without glomerulonephritis

No	Age/gender	Clinical manifestations	Elevated sCr (μmol/L)^a^	Proteinuria (g/day)^a^	Hematuria (/μL)^a^	Anemia (g/L)^a^	ANCA (AU/mL)^a^	Tubulointerstitial lesions	Glomerular lesions	Treatment^b^	Outcome	Author (year)
Case 1	78/M	Fever, abdominal distension, anorexia, oliguria, edema, dyspnea, hypertension	481	0.38	2.3	71	MPO 184	Interstitial edema and fibrosis, intense inflammatory infiltrates, TBM thickening, tubular atrophy	Global or segmental sclerosis	CS, CTX, Rtx	Remitted	Local case
Case 2	64/M	Fever, fatigue, myalgia, arthralgia, edema, myasthenia, interstitial lung lesions	159	0.34	54.4	113	MPO 239	Diffuse inflammatory cell infiltration in the interstitium, destructed tubules	Normal	CS, CTX	Remitted	Local case
Case 3	67/F	Hypertension	113	0.12	2.3	142	MPO 39.4	Interstitial edema and fibrosis, intense inflammatory infiltrates, TBM thickening, tubular atrophy, strong staining of IgA, κ-chain and λ-chain in tubular areas	Slight proliferation	CS, CTX	Remitted	Local case
1	83/F	Fever, anorexia	+	-	-	+	MPO	Peritubular capillaritis, TBM^d^ lysis, tubulitis	Neutrophils in glomerular capillary loops	NA	NA	Nakabayashi (2009) [1]
2	73/M	Fever, cough, sputum, myalgia, arthralgia, anorexia, weight loss	-	-	-	-	MPO	Peritubular capillaritis, TBM lysis, tubulitis	Neutrophils in glomerular capillary loops	CS	NA	Nakabayashi (2009) [1]
3	62/F	Fever, cough, myalgia, arthralgia, peripheral neuropathy, edema, anorexia, weight loss	-	-	-	+	MPO	Peritubular capillaritis, TBM lysis, tubulitis	Neutrophils in glomerular capillary loops, global sclerosis	NA	NA	Nakabayashi (2009) [1]
4	74/F	Fever, edema, purpura	+	0.3 g/day	1–4/HPF	+	MPO	Tubulitis, peritubular capillaritis, fibrinoid vasculitis, diffuse infiltration of inflammatory cells	Collapsing capillary loops	CS, CTX	Remitted	Kasahara (2014) [2]
5	45/F	Arthralgia; recurrent sinusitis	+	3.3 g/day	Microscopic	-	MPO	Acute interstitial nephritis, mild interstitial fibrosis, tubular atrophy, mild focal hyaline arteriosclerosis	Normal	CS, AZA, CsA, MMF, Rtx	Noneffective	Plafkin (2019) [3]
6	77/F	Lethargy	+	2.3 g/day	Microscopic	-	PR3	Acute tubulointerstitial nephritis with an interstitial granuloma	Minor glomerular abnormalities	CS	Relapse 1 year later	Guo (2020) [4]
7	17/M	Edema, fatigue, anuria; allergic rhinitis, allergic asthma	+	1 +	228/HPF	-	MPO	Granulomatous inflammation, massive destruction of tubules, extensive interstitial infiltration of inflammatory cells	Diffuse destruction of glomerular structure with crescents and severely ruptured Bowman’s capsules	PE, CS, Rtx	ANCA negative, but hemodialysis	Lin (2019) [5]
8	59/F	Fever, fatigue; bronchial asthma	+	1.1 g/day	Numerous	+	MPO	Slight fibrosis and marked infiltration of eosinophils, hyalinized arteries and arterioles	Segmental necrotizing lesions (2/19 glomeruli), intraluminal eosinophils and neutrophils	CS, CTX	Remitted	Hirohama (2012) [6]
9	78/M	Lethargy, cough, breathlessness, purpuric rash, arthropathy	+	-	2 +	-	+	Pronounced interstitial edema, prominent aggregates of neutrophils, capillary endothelial cell damage with extravasation of RBCs	Normal	CS, CTX	Improved, but anuric	Banerjee (2001) [7]
10	70/F	Fatigue, anorexia	+	1.32 g/day	1–4/HPF	-	MPO	Tubulointerstitial injury, diffuse infiltration of inflammatory cells, mild to moderate fibrosis, peritubular capillaritis and leukocyte casts	Global sclerosis (3/30 glomeruli)	CS	Remitted	Morimoto (2021) [8]
11	27/M	Anasarca, sensory neuropathy, recurrent upper airway congestion, epistaxis	+	4.2 g/day	3 +	+	-	Granulomatous interstitial nephritis, expansion of the interstitium, inflammatory infiltrates, edema with granulomas	Normal	CS, CTX	Remitted	Tiewsoh (2020) [9]
12	44/M	Malaise, edema, hypertension	+	4 +	20–25/HPF	_	MPO	Diffuse infiltrate of mononuclear cells in the interstitium and tubular epithelium	Normal	CS, CTX	Remitted	Wen (2006) [10]
13	70/F	Fever, backache, arthralgia, foot drop; interstitial lung disease	+	PCR^e^ 907.4 mg/g	10–19/HPF	+	MPO and PR3	Segmental mesangial cell proliferation, focal atrophy and loss of tubules, mononuclear cell infiltration with fibrosis, widely effaced foot processes	Normal	CS, AZA	Remitted	Kim (2020) [11]
14	63/F	Arthralgia, solid mass in the thorax, Raynaud’s phenomenon, amyotrophy	-	25 mg/dL	Numerous	-	PR3	Tubular atrophy, foci of fibrosis, and dense inflammation	Minimal nonspecific proliferation	CS, CTX	Remitted	Ernam (2003) [12]
15	75/M	Asthenia, anorexia, weight loss	+	1.4 g/day	1 +	+	MPO	Interstitium fibro-edema, diffuse inflammatory infiltrate, epithelial necrosis and atrophy of tubules, significant tubulitis	Slight mesangial expansion	CS	Remitted	Hassani (2013) [13]

^a^The varied descriptions for the proteinuria or hematuria were due to various standards of local cases and the cited studies

^b^Abbreviations: *NA*, not applicable; *CS*, corticosteroids; *CTX*, cyclophosphamide; *AZA*, azathioprine; *CsA*, cyclosporine; *MMF*, mycophenolic acid; *Rtx*, rituximab; *PE*, plasma exchange; *TBM*, tubular basement membrane; *PCR*, spot urine protein/creatinine ratio; *sCr*, serum creatine; *MPO*, myeloperoxidase-antineutrophil cytoplasmic antibody; *PR3*, proteinase 3-antineutrophil cytoplasmic antibody

**Table 2:** Summary of 6 local cases with ANCA-associated vasculitis causing typical glomerulonephritis

No	Age/gender	Clinical manifestations	Serum creatine (μmol/L)	24hUP^a^	Hematuria^b^	Hemoglobin (g/L)	ANCA	Treatment^c^	Outcome
1	75/M	Anorexia	258	0.63	445.4	101	232	CS, CTX	Remitted
2	72/M	Fever, dyspnea, headache, and fatigue	571	0.15	3	62	229	CS, CTX, PE	ESRD
3	61/M	Asthenia, anorexia	211	1.66	280.8	108	224	CS, CTX	Remitted
4	61/M	Fever, dyspnea, edema	514	0.96	192.4	111	239	CS, CTX	Remitted
5	67/F	Urination discomfort	164	4.42	351.1	113	171	CS, CTX, PE, Rtx	Remitted
6	76/F	Oliguria, edema	123	0.04	194.2	114	166	CS, CTX	Remitted

Abbreviations: *CS*, corticosteroids; *CTX*, cyclophosphamide; *PE*, plasma exchange; *Rtx*, rituximab; *TBM*, tubular basement membrane; *GBM*, anti-glomerular basement membrane; *sCr*, serum creatine; *24hUP*, 24-h urinary protein; *Hgb*, hemoglobin; *MPO*, myeloperoxidase-antineutrophil cytoplasmic antibody; *ESRD*, end-stage renal disease

## Case reports

### Case 1

**Fig. 2:**
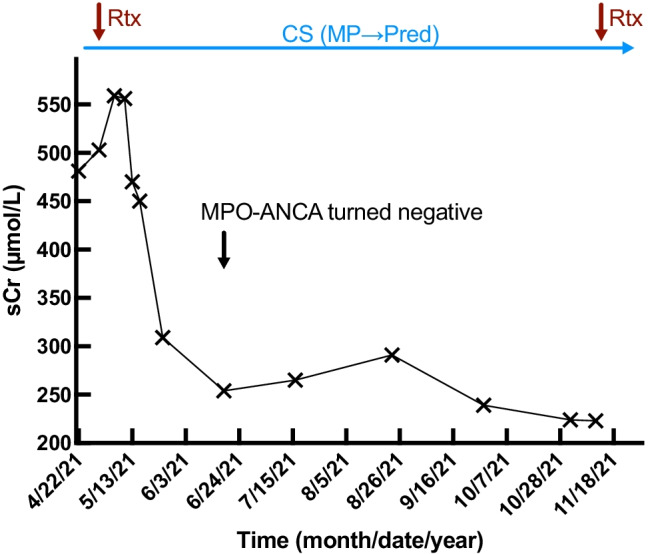
Treatment response of the local case 1. The 78-year-old patient received intravenous methylprednisolone infusion (80 mg/day for three days), followed by oral prednisone (60 mg/day, 1.0 mg/kg/day). Rituximab infusions were at an interval of 6 months (1 g and 0.5 g, separately). Two months after the initial treatment, ANCA turned negative, and at the six months follow-up, serum creatine decreased to 223 μmol/L. Rtx, rituximab; CS, corticosteroids; MP, methylprednisolone; Pred, prednisone; MPO-ANCA, antineutrophil cytoplasmic antibody specific for myeloperoxidase; sCr, serum creatine

A 78-year-old male was admitted to PUMCH on April 21, 2021, with the chief complaint of impaired renal function for six months, low-grade fever, abdominal distension, and loss of appetite for three months. Soon, a deterioration of renal function (serum creatine, sCr, notably rising from 231 to 449 μmol/L in 29 days) and progressive anemia (hemoglobin, Hgb, 106 to 67 g/L in 10 days) was noted, followed by oliguria, edema, dyspnea, and hypertension. The patient also had a past medical history of subtotal gastrectomy in 1959 and gastrointestinal bleeding in 2018. On physical examination, his blood pressure was 166/78 mmHg. Routine lab tests revealed remarkable anemia (hemoglobin 71 g/L), significant renal dysfunction (sCr 481 μmol/L), and elevated inflammatory markers. His erythrocyte sedimentation rate (ESR) was 45 mm/h, and his high-sensitivity C-reactive protein (hsCRP) was 17.18 mg/L. Urinalysis showed mild proteinuria 0.38 g/24 h without hematuria. He also demonstrated positivity for p-ANCA 1:20 (immunofluorescence method), anti-MPO-ANCA 184 AU/mL (chemiluminescence method), and elevated IgG4 with a value of 10,700 mg/L (80–1400). The patient was negative for ANAs and anti-glomerular basement membrane (GBM) antibody. Immunofluorescent (IF) staining was negative, and no immune-complex deposition was observed in the glomerulus by electron microscopy (EM). The light microscope (LM) analysis showed a total of 16 glomeruli, with global sclerosis and segmental sclerosis glomeruli of 56.3% and 12.5%, respectively, without significant proliferation. The interstitium was infiltrated by lymphocytes, monocytes, scattered eosinophils, neutrophils, and plasma cells, with edema and fibrosis and negative for IgG4 staining. Diagnosed as AAV-associated interstitial nephritis, the patient was treated with intravenous methylprednisolone (80 mg/day for three days), followed by oral prednisone (60 mg/day and tapering), with infused rituximab (Rtx) (1 g) on May 1. The patient responded well to the treatment. His renal function showed significant improvement, and the ANCA turned negative at two months. At the six-month follow-up, his sCr was 223 μmol/L (Fig. 2).

### Case 2

A 64-year-old male was admitted to PUMCH on December 9, 2016, due to progressive fatigue, myalgia, arthralgia, and edema of the lower extremities for one month and fever (38.5 ℃) for 12 days. On physical examination, his blood pressure was 126/60 mmHg. Routine lab tests revealed slight anemia (hemoglobin 113 g/L), elevated inflammatory markers (WBC 14.59 × 10^9^/L, ESR 97 mm/h), and impaired renal function (sCr 159 μmol/L). Urinalysis was positive for red blood cells (RBCs) 54.4/μL and negative for protein, with 24-h urinary protein (24hUP) of 0.34 g. Immunological studies revealed positive c-ANCA IF 1:80, anti-PR3-ANCA 132 RU/mL (enzyme-linked immunosorbent assay), and MPO-ANCA > 200 AU/mL. His chest CT revealed multiple ground-glass patches on bilateral lungs. His renal biopsy showed predominantly interstitial nephritis. On LM, there was no obvious abnormality in the glomerulus with diffuse interstitial inflammatory cell infiltration and the destruction of renal tubules. Some renal tubular epithelial cells were slightly denatured. There was no sign of necrotizing glomerular vasculitis. No deposits of immunoglobulins or complements were displayed by IF with only one sclerotic glomerulus on EM. For this patient with AAV-associated acute interstitial nephritis complicated with possible pulmonary involvement, oral prednisone (60 mg/day, 1.0 mg/kg/day) and oral cyclophosphamide (50 mg/day) were prescribed. At the follow-ups, the patient reported a continual resolution of symptoms. Five months later, his sCr returned to 86 μmol/L, and his ANCA turned negative.

### Case 3

A 67-year-old female was admitted to PUMCH on May 6, 2021, because of hypertension for two months. The patient first noticed a blood pressure of 160/95 mmHg, and later lab tests showed an elevated sCr of 108 μmol/L two months ago without other reported clinical manifestations. Her past medical history was significant for hyperlipidemia, uveitis, and senile macular degeneration. Both proteinuria and hematuria were negative in routine urinary analysis, with 24hUP 0.12 g. Impaired renal function (sCr 113 μmol/L), positive p-ANCA IF 1:10, and anti-MPO-ANCA 39.4 AU/mL were observed. Her renal biopsy showed interstitial nephritis. On LM, occasional segmental mesangial cell proliferation and mesangial matrix expansion, vacuolar degeneration, and brush border abscission were present in renal tubular epithelial cells. In addition to lymphocyte infiltration in several renal tubules, we observed diffuse interstitial edema, accompanied by a large number of lymphocytes, monocytes, scattered eosinophils, neutrophils, and plasma cells. On IF, there was no positive deposition in the glomerulus, with an occasional proliferation of mesangial cells and mesangial matrix and epithelial foot process fusion by EM. We also noticed some GBM degeneration and shrinkage without electronic density or other abnormal deposits.

A diagnosis of AAV-associated chronic interstitial nephritis was made. Oral prednisone (55 mg/day, 0.8 mg/kg/day) was given, accompanied by oral cyclophosphamide (50 mg/day). During the follow-up, the patient reported a continual resolution of symptoms. Four months later, her sCr level had improved (101 μmol/L), and ANCA turned negative.

### Comparison of AAV-associated interstitial nephritis without glomerulonephritis and AAV-associated typical glomerulonephritis

Six cases of AAV-associated typical glomerulonephritis diagnosed at PUMCH were selected as the control group. The patients with nephritis showed comparable sCr, 24hUP, urinary RBCs, hemoglobin, and MPO titer. The average sCr, 24hUP, urinary RBCs, hemoglobin, and MPO tilter were 251.0 ± 115.8 and 306.8 ± 77.1 μmol/L (*P* = 0.709), 0.28 ± 0.08 and 1.31 ± 0.67 g/24 h (*P* = 0.184), 19.7 ± 17.4 and 244.5 ± 62.3/μL (*P* = 0.014), 108.7 ± 20.6 and 101.5 ± 8.1 g/L (*P* = 0.770), and 154.1 ± 59.5 and 210.2 ± 13.3 AU/mL (*P* = 0.447) in AAV-associated interstitial nephritis without glomerulonephritis and the control group, respectively.

**Fig. 3:**
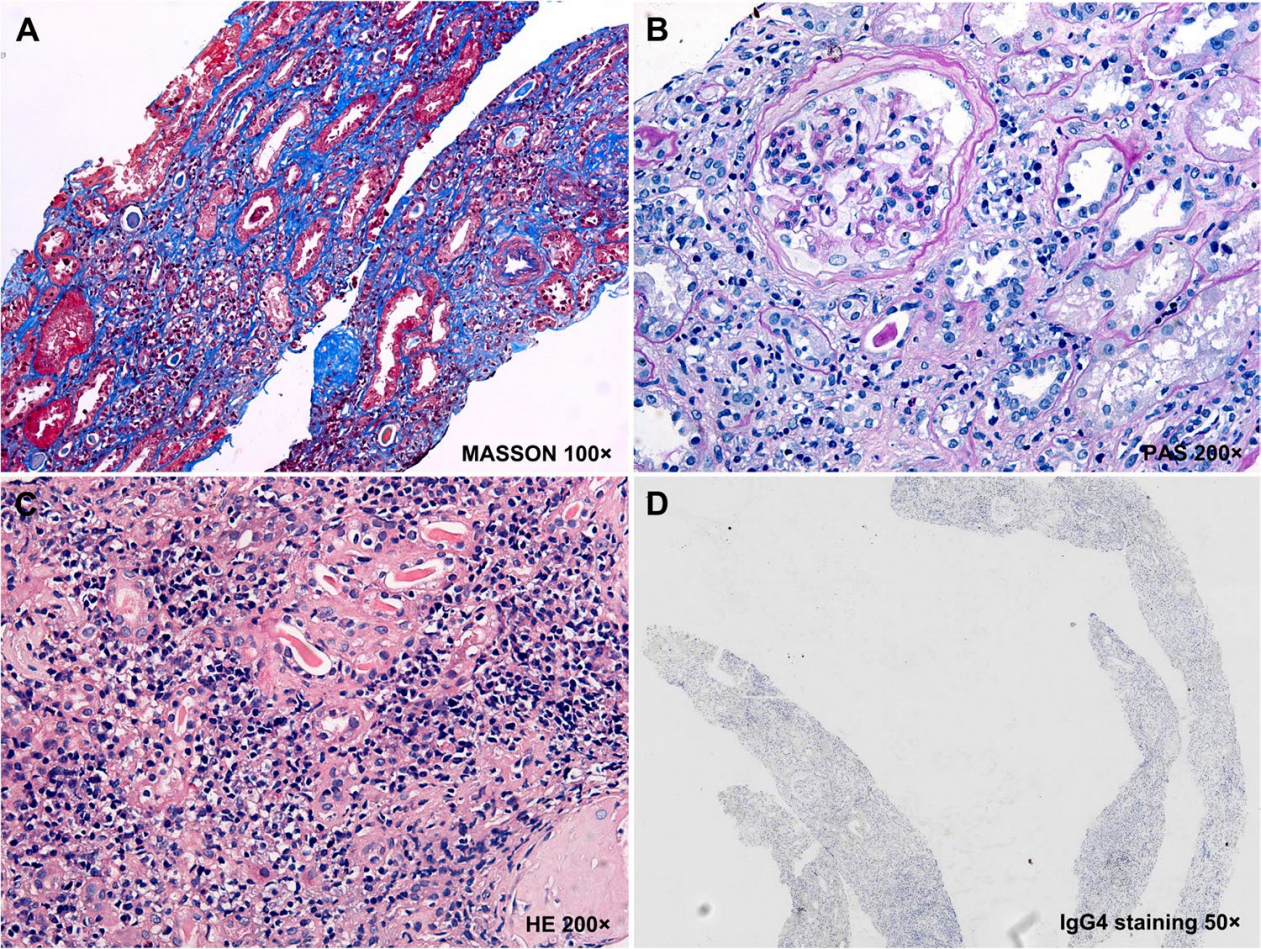
Pathologic findings in kidney tissues from local cases of AAV with interstitial nephritis. (**A**) An overall sight showed intense inflammatory infiltrates and mild fibrosis in the interstitial area (MASSON 100 ×). (**B**) Inflammatory infiltrates in the interstitium without evident glomerulonephritis was noted (PAS 200 ×). (**C**) Diffuse infiltration of inflammatory cells was present in the interstitium (HE 200 ×). (**D**) IgG4 staining showed negative results (IgG4 staining 50 ×). AAV, antineutrophil cytoplasmic antibody–associated vasculitis; HE, hematoxylin and eosin staining; MASSON: Masson’s trichrome staining; PAS: periodic acid-Schiff staining

Upon renal pathology, AAV-associated glomerulonephritis usually showed cellular crescents and cellular fibrous crescents, segmental mesangial cell proliferation and increased mesangial matrix, GBM denaturation, and sometimes rupture of Bowman’s capsules. Their interstitial lesions usually showed mild fibrosis and diffuse infiltration of mononuclear inflammatory cells. In contrast, AAV causing interstitial nephritis alone often showed interstitial edema and fibrosis, intense inflammatory infiltrates, tubular basement membrane thickening, and tubular atrophy (Tables 1 and 2, Fig. 3).

For treatment, most of these cases received corticosteroids and cyclophosphamide as the first choice. Plasma exchange was applied in two cases. Notably, Rtx was used in both groups (1/3 in AAV-associated interstitial nephritis and 1/6 in AAV-associated typical glomerulonephritis). Almost all of the local cases achieved remission in follow-ups, except one case that reached ESRD.

### Literature research

We screened 154 studies, with 15 eligible cases in 13 studies [10–22] (Table 1). The reported patients’ average age was 61.1 years old, and 60% of them were female with nonspecific symptoms of fever (40%), arthralgias (40%), edema (33.3%), anorexia, fatigue, weight loss, neuropathy, purpura, or epistaxis. Respiratory symptoms or comorbidities of asthma or interstitial lung diseases were common (53.3%). Most of them had elevated sCr levels (80%), proteinuria (73.3%), hematuria (73.3%), ANCA or MPO positivity (66.7%), and anemia (46.7%). A few of them had massive proteinuria (one patient had more than 3.5 g/day) and numerous urine RBCs (13.3%, Table 1). Renal pathology showed renal interstitial nephritis with mild or no glomerular lesions. Diffuse infiltration of inflammatory cells, interstitial fibrosis, interstitial edema, tubulitis, and vasculitis were common. Other lesions in the interstitial areas included tubular atrophy, granulomatous injury, or hyaline arteriosclerosis. Glomerular lesions were absent or mild.

Most of the cases received medium to a high dose of corticosteroids as the first choice with or without immunosuppressants. Cyclophosphamide remained the most common immunosuppressant, with the alternative being azathioprine, cyclosporine, or mycophenolic acid. Rtx or plasma exchange was applied recently. The patients’ responses varied with remitted (66.7%), dialysis depending (16.7%), and no effectiveness (8.3%).

## Discussion

AAV-associated interstitial nephritis is a rare complication of AAV without universally accepted diagnostic criteria, which leads to several challenges in clinical management. In this study, we reported three cases of these patients and reviewed the literature. AAV-associated interstitial nephritis tended to occur in aged patients with nonspecific symptoms and positive MPO-ANCA. Characterized by isolated interstitial inflammation and tubulitis, patients typically responded well to immunotherapy. Compared with AAV-associated typical glomerulonephritis, these cases showed significantly fewer urinary RBCs and tended to exhibit lower levels of sCr, 24hUP, and MPO tilter. Moreover, AAV-associated interstitial nephritis without glomerulonephritis tended to exhibit lower levels of sCr, 24hUP, and MPO titers with no significance.

In addition to the diagnostic value in AAV, ANCAs also play a pathogenic role in disease development. All three cases tested positive for MPO-ANCA, consistent with the 15 cases in the literature (66.7%), which was more often associated with renal manifestations than PR3-ANCA [4] and correlated with AAV disease activity and risk of relapse [23, 24]. Once primed by proinflammatory cytokines, MPO-ANCAs bind their antigens and trigger neutrophil degranulation, leading to the release of reactive oxygen species and lytic enzymes developing into consequent endothelial damage and small-vessel vasculitis [25].

Renal biopsies in AAV typically reveal pauci-immune necrotizing crescentic glomerulonephritis, which is consistent with our six local cases with AAV-associated typical glomerulonephritis. In studies from the National Institutes of Health (NIH) in the USA, glomerulonephritis developed in 77 to 85% of patients, usually within the first two years of disease onset [26]. Interstitial nephritis is often accompanied by glomerulitis, and isolated interstitial nephritis without glomerulitis is rare [17, 27, 28]. Unlike the urgent therapy before renal biopsy for patients with typical glomerular involvement and ANCA positivity, for elderly patients who present with only nonspecific symptoms, renal pathology is of increased value for accurate diagnosis and subsequent medical decisions. The recognition of the atypical variant of AAV is also crucial.

A detailed mechanism for AAV-associated interstitial nephritis remains unknown [19], with limited clues from the literature. Peritubular capillaritis is common in AAV with glomerular capillaritis, vasculitis in vessels, and extravascular granuloma formation [18]. This result indicated that the loss of CD34 vascular endothelial markers occurs in the early phase of the disease [19] with infiltration of neutrophils and mononuclear cells into tubular epithelial cells, which was associated with tubular basement membrane lysis. A few AAV patients with acute interstitial nephritis progressed to crescentic glomerulonephritis. Their confounding factors were cimetidine treatment and other unknown mechanisms [10, 18, 22]. Further research is required to reveal the exact pathogenesis of AAV-associated interstitial nephritis.

The therapy for AAV mainly incorporates glucocorticoids, immunosuppressants, and Rtx [29]. Due to the scarcity of patients, there is no recommendation for AAV-associated interstitial nephritis. A moderate dose of corticosteroids is a common choice to avoid severe infection in aged patients [30] [31]. Two of our reported cases had good responses to corticosteroids combined with cyclophosphamide. Their prognosis is better than that of necrotizing crescentic AAV reported previously [12, 27]. Plasma exchange is more often used in AAV-associated glomerulonephritis. An alternative therapy was Rtx, a monoclonal antibody targeting the CD20 antigen; combined with glucocorticoids, it achieved satisfying efficacy consistent with common AAV [27, 29, 32, 33]. In our local cases, Rtx was used both in AAV causing merely interstitial nephritis and AAV-associated glomerulonephritis. More clinical studies are required to explore the best therapy for AAV-associated interstitial nephritis.

### Limitations

The sample size of the reported cases was limited because of the rare phenotype of AAV. The exact pathogenesis and mechanism of AAV-associated interstitial nephritis were not investigated. Future research needs to recruit more cases and extend more details of this variant of AAV for better medical care.

## Conclusions

AAV-associated interstitial nephritis in aged patients with nonspecific symptoms is usually associated with MPO-ANCA. The pathogenic lesions are characterized by diffuse infiltration of inflammatory cells, interstitial fibrosis, edema, and tubulitis. Immunosuppressive therapy showed good efficacy in these patients.

## References

[1] Seo P, Stone JH (2004). The antineutrophil cytoplasmic antibody-associated vasculitides. Am J Med.

[2] Jennette JC, Falk RJ (1997). Small-vessel vasculitis. N Engl J Med.

[3] Kitching AR, Anders HJ, Basu N, Brouwer E, Gordon J, Jayne DR, Kullman J, Lyons PA, Merkel PA, Savage COS, Specks U, Kain R (2020). ANCA-associated vasculitis Nat Rev Dis Primers.

[4] Lionaki S, Blyth ER, Hogan SL, Hu Y, Senior BA, Jennette CE, Nachman PH, Jennette JC, Falk RJ (2012). Classification of antineutrophil cytoplasmic autoantibody vasculitides: the role of antineutrophil cytoplasmic autoantibody specificity for myeloperoxidase or proteinase 3 in disease recognition and prognosis. Arthritis Rheum.

[5] Haas M, Eustace JA (2004). Immune complex deposits in ANCA-associated crescentic glomerulonephritis: a study of 126 cases. Kidney Int.

[6] Eisenberger U, Fakhouri F, Vanhille P, Beaufils H, Mahr A, Guillevin L, Lesavre P, Noel LH (2005). ANCA-negative pauci-immune renal vasculitis: histology and outcome. Nephrol Dial Transplant.

[7] Hauer HA, Bajema IM, van Houwelingen HC, Ferrario F, Noel LH, Waldherr R, Jayne DR, Rasmussen N, Bruijn JA, Hagen EC, European Vasculitis Study G (2002). Renal histology in ANCA-associated vasculitis: differences between diagnostic and serologic subgroups. Kidney Int.

[8] Jennette JC, Falk RJ (1994). The pathology of vasculitis involving the kidney. Am J Kidney Dis.

[9] Sakai N, Wada T, Shimizu M, Segawa C, Furuichi K, Kobayashi K, Yokoyama H (1999). Tubulointerstitial nephritis with anti-neutrophil cytoplasmic antibody following indomethacin treatment. Nephrol Dial Transplant.

[10] Banerjee A, McKane W, Thiru S, Farrington K (2001). Wegener’s granulomatosis presenting as acute suppurative interstitial nephritis. J Clin Pathol.

[11] Ernam D, Atikcan S, Yilmaz A, Atalay F, Demirag F, Memis L (2003). An unusual renal presentation of Wegener’s granulomatosis. Tuberk Toraks.

[12] Guo W, Woo KT, Choo JC, Tan PH, Lim CC (2020). Granulomatosis with polyangiitis and acute tubulointerstitial nephritis in the absence of glomerulonephritis. Am J Med.

[13] Hassani K, Hamzi AM, Hassani M, Benyahia M (2013). Acute tubulo-interstitial nephritis with positive anti-neutrophil cytoplasmic antibodies. Arab J Nephrol Transplant.

[14] Hirohama D, Hoshino J, Sumida K, Hasegawa E, Hiramatsu R, Yamanouchi M, Hayami N, Suwabe T, Sawa N, Takemoto F, Ubara Y, Hara S, Ohashi K, Takaichi K (2012). Churg-Strauss syndrome presenting with acute renal insufficiency accompanied by eosinophilic tubulointerstitial nephritis. Internal medicine (Tokyo, Japan).

[15] Kasahara H, Hiroyuki N, Shinohara M, Koike T (2014). AP-VAS 2012 case report: an atypical case of microscopic polyangiitis presenting with acute tubulointerstitial nephritis without glomerular change. CEN Case Rep.

[16] Kim SH, Kim H-R, Lee S-H, Min HK (2021). Tubulointerstitial nephritis without glomerular crescent formation as an underestimated subgroup of renal involvement among microscopic polyangiitis patients: a case report. Clin Case Rep.

[17] Lin ZS, Liu XL, Cui Z, Wang SX, Yu F, Zhou FD, Zhao MH (2019). Acute tubulointerstitial nephritis with germinal centers in antineutrophil cytoplasmic antibody-associated vasculitis: a case report and literature review. Medicine (Baltimore).

[18] Morimoto K, Kanzaki G, Niikura T, Koike K, Matsuo N, Maruyama Y, Tsuboi N, Yokoo T (2021). Acute tubulointerstitial nephritis associated with antineutrophil cytoplasmic antibody following cimetidine treatment: a case report. BMC Nephrol.

[19] Nakabayashi K, Sumiishi A, Sano K, Fujioka Y, Yamada A, Karube M, Koji H, Arimura Y, Nagasawa T (2009). Tubulointerstitial nephritis without glomerular lesions in three patients with myeloperoxidase-ANCA-associated vasculitis. Clin Exp Nephrol.

[20] Plafkin C, Zhong W, Singh T (2019). ANCA vasculitis presenting with acute interstitial nephritis without glomerular involvement. Clin Nephrol Case Stud.

[21] Tiewsoh I, Dey B, Lyngdoh M, Lynrah K, Synrem E, Mitra A (2020). Granulomatous interstitial nephritis in granulomatosis with polyangiitis mimicking leprosy: a case report. J Family Med Prim Care.

[22] Wen Y-K, Chen M-L (2006). Transformation from tubulointerstitial nephritis to crescentic glomerulonephritis: an unusual presentation of ANCA-associated renal vasculitis. Ren Fail.

[23] Arimura Y, Kawashima S, Yoshihara K, Komagata Y, Kaname S, Yamada A (2013). The role of myeloperoxidase and myeloperoxidase-antineutrophil cytoplasmic antibodies (MPO-ANCAs) in the pathogenesis of human MPO-ANCA-associated glomerulonephritis. Clin Exp Nephrol.

[24] Kallenberg CG (2011). Pathogenesis of ANCA-associated vasculitides. Ann Rheum Dis.

[25] Rowaiye OO, Kusztal M, Klinger M (2015). The kidneys and ANCA-associated vasculitis: from pathogenesis to diagnosis. Clin Kidney J.

[26] Hoffman GS, Kerr GS, Leavitt RY, Hallahan CW, Lebovics RS, Travis WD, Rottem M, Fauci AS (1992). Wegener granulomatosis: an analysis of 158 patients. Ann Intern Med.

[27] Geetha D, Jefferson JA (2020). ANCA-associated vasculitis: core curriculum 2020. Am J Kidney Dis.

[28] Schonermarck U, Schirren CA, Mistry-Burchardi N, Weiss M, Eichhorn P, Samtleben W (2005). Interstitial nephritis and high titers of PR3-ANCA: an unusual manifestation of ANCA-associated disease. Clin Nephrol.

[29] Wallace ZS, Miloslavsky EM (2020). Management of ANCA associated vasculitis. BMJ.

[30] Moledina DG, Perazella MA (2017). Drug-induced acute interstitial nephritis. Clin J Am Soc Nephrol.

[31] Rovin BH, Adler SG, Barratt J, Bridoux F, Burdge KA, Chan TM, Cook HT, Fervenza FC, Gibson KL, Glassock RJ, Jayne DRW, Jha V, Liew A, Liu ZH, Mejia-Vilet JM, Nester CM, Radhakrishnan J, Rave EM, Reich HN, Ronco P, Sanders JF, Sethi S, Suzuki Y, Tang SCW, Tesar V, Vivarelli M, Wetzels JFM, Lytvyn L, Craig JC, Tunnicliffe DJ, Howell M, Tonelli MA, Cheung M, Earley A, Floege J (2021). Executive summary of the KDIGO 2021 Guideline for the Management of Glomerular Diseases. Kidney Int.

[32] Hassan RI, Gaffo AL (2017). Rituximab in ANCA-associated vasculitis. Curr Rheumatol Rep.

[33] Smith RM, Jones RB, Specks U, Bond S, Nodale M, Aljayyousi R, Andrews J, Bruchfeld A, Camilleri B, Carette S, Cheung CK, Derebail V, Doulton T, Forbess L, Fujimoto S, Furuta S, Gewurz-Singer O, Harper L, Ito-Ihara T, Khalidi N, Klocke R, Koening C, Komagata Y, Langford C, Lanyon P, Luqmani RA, Makino H, McAlear CA, Monach P, Moreland LW, Mynard K, Nachman P, Pagnoux C, Pearce F, Peh CA, Pusey C, Ranganathan D, Rhee RL, Spiera R, Sreih AG, Tesar V, Walters G, Weisman MH, Wroe C, Merkel PA, Jayne D, coinvestigators R, co-investigators R,  (2020). Rituximab as therapy to induce remission after relapse in ANCA-associated vasculitis. Ann Rheum Dis.

